# Large-scale genetic variation of the symbiosis-required megaplasmid pSymA revealed by comparative genomic analysis of *Sinorhizobium meliloti *natural strains

**DOI:** 10.1186/1471-2164-6-158

**Published:** 2005-11-10

**Authors:** Elisa Giuntini, Alessio Mengoni, Carlotta De Filippo, Duccio Cavalieri, Nadia Aubin-Horth, Christian R Landry, Anke Becker, Marco Bazzicalupo

**Affiliations:** 1 Dipartimento di Biologia Animale e Genetica, Università di Firenze, via Romana 17, I-50125 Firenze, Italy; 2 Dipartimento di Farmacologia, Università di Firenze, Viale Pieraccini 6, 50139 Firenze, Italy; 3 Bauer Center for Genomics Research, Harvard University, 7 Divinity Avenue, Cambridge, Massachusetts, 02138, USA; 4 Department of Organismic and Evolutionary Biology, Harvard University, 16 Divinity Avenue, Cambridge, Massachusetts, 02138, USA; 5 Lehrstuhl fur Genetik, Universitat Bielefeld, 33594 Bielefeld, Germany

## Abstract

**Background:**

*Sinorhizobium meliloti *is a soil bacterium that forms nitrogen-fixing nodules on the roots of leguminous plants such as alfalfa (*Medicago sativa*). This species occupies different ecological niches, being present as a free-living soil bacterium and as a symbiont of plant root nodules. The genome of the type strain Rm 1021 contains one chromosome and two megaplasmids for a total genome size of 6 Mb. We applied comparative genomic hybridisation (CGH) on an oligonucleotide microarrays to estimate genetic variation at the genomic level in four natural strains, two isolated from Italian agricultural soil and two from desert soil in the Aral Sea region.

**Results:**

From 4.6 to 5.7 percent of the genes showed a pattern of hybridisation concordant with deletion, nucleotide divergence or ORF duplication when compared to the type strain Rm 1021. A large number of these polymorphisms were confirmed by sequencing and Southern blot. A statistically significant fraction of these variable genes was found on the pSymA megaplasmid and grouped in clusters. These variable genes were found to be mainly transposases or genes with unknown function.

**Conclusion:**

The obtained results allow to conclude that the symbiosis-required megaplasmid pSymA can be considered the major hot-spot for intra-specific differentiation in *S. meliloti*.

## Background

Environmental bacteria are free-living bacteria colonising soil and water. Most of these species are involved in key steps of the biogeochemical cycles of elements such as nitrogen, sulphur, iron, phosphorus and carbon [1]. One of the genomic features of environmental bacteria, and particularly of those belonging to the α-proteobacteria subdivision, is the presence of large genomes of several megabases, consisting of many replicons of similar size, whereas pathogenic and parasitic bacterial genomes often consist of a single replicon. In particular, many of the symbiotic nitrogen-fixing bacteria are characterised by the presence of multiple megaplasmids [2]. In an evolutionary perspective, plasmids have been shown to contribute to symbiosis, pathogenesis and colonisation of new environments, providing resistance to antibiotics or the ability to use specific carbon sources [3-5]. Because megaplasmids can be as large as bacterial genomes and are often not conjugative, their evolutionary dynamics may be closer to that of a real chromosome [2]. Therefore, the role of such megaplasmids in adaptation and consequently their genomic dynamics in the bacterial species is particularly intriguing in the perspective of complex, multi-replicon genome evolution.

Comparative genomic hybridisation (CGH) is a powerful methodology which relies on microarray genome-wide comparison of DNA from different organisms or cells [6-9]. In the field of microbiology, where the number of sequenced species is over 200, CGH has been applied to investigate genomic variation in a certain number of bacterial strains, mainly human pathogens, in order to relate genomic feature to virulence and host adaptation [10-24]. These studies showed that the main sources of variation within bacterial genomes were often duplications or deletions of large DNA fragments. Up to now, most of these studies were performed on species whose genome consist of one replicon and therefore very limited information is available about the genome-scale polymorphism in bacterial species with complex multi-replicon genomes [23]. Here we address this issue in the bacterium *Sinorhizobium meliloti*.

*Sinorhizobium meliloti *is a soil bacterium that forms nitrogen-fixing nodules on the roots of leguminous plants such alfalfa (*Medicago sativa*). It belongs to the *Rhizobiales *group of the α-Proteobacteria subdivision, together with important human pathogens such as *Bartonella *and *Brucella*, and with several plant-associated bacteria of major agricultural importance, such as *Agrobacterium*, *Ochrobactrum*, *Bradyrhizobium*, *Mesorhizobium *and *Rhizobium *[2]. *S. meliloti *is distributed world-wide and is present in many soil types, both in association with legumes or in a free-living form [25]. This species is a model species to study plant-bacteria interactions and in particular legume-rhizobia symbiosis and symbiotic nitrogen-fixation. Its genome contains 6206 ORFs distributed in three replicons, one chromosome of 3.6 Mbp and two megaplasmids, 1.3 Mbp and 1.7 Mbp in size [26-30]. The smallest of the megaplasmids, called either pSymA, pNod-Nif, or pRmeSU47a, contains 1293 ORFs, including many of the genes involved in root nodule formation (*nod*) and nitrogen fixation (*nif*) [28,31,32]. The other megaplasmid, pSymB, contains 1570 ORFs and carries genes encoding solute uptake systems, genes involved in polysaccharide biosynthesis and in catabolic activities [29]. Finally, most of 3342 predicted ORFs of the chromosome code for proteins involved in transport and degradation of amino-acids and peptides, as well as sugar metabolism [30].

Previous studies using molecular markers showed that natural populations of rhizobia, and in particular of *S. meliloti*, exhibit high levels of genetic polymorphism [33-38]. These natural strains also harbour a high number of different mobile genetic elements such as insertion sequences (IS), transposons and bacterial mobile introns [39-41]. However, which functional genes are variable in natural populations contributing to ecological adaptations remains to be fully investigated. Moreover, how the evolutionary dynamics of the diverse replicons differ is still unknown.

To address these questions, genomic DNA of four strains of *S. meliloti*, previously isolated from agricultural Italian soil and from soil around the Aral Sea region, were compared with the sequenced laboratory strain Rm1021 on a full-genome *S. meliloti *microarray [42].

## Results

### Overall results

Four strains, two isolates from soils in the Aral Sea region and two from Northern Italy soil, were compared by whole genome hybridisation with type strain Rm 1021. Four slides with three copies of each ORF were used for each comparison and the results were analysed as described in Methods. Genes were considered to be variable if a statistically significant difference (*P *< 0.001) in hybridisation intensity was detected between the type and the strain under comparison. The fraction of variable genes detected with comparative genomic hybridisation (CGH) on the microarray containing oligonucleotide probes for all currently predicted protein-coding genes of strain Rm1021, ranged from 4.6 to 6.5% (Table 2). In particular, strain BL225C showed the highest number of variable genes (401), while strain AK58 displayed the lowest number (287). The majority of variable genes (77–94%) showed decreased hybridisation intensity (*Log2-ratio *> 0) of the natural isolate versus the Rm 1021 strain, suggesting deletion or nucleotide divergence in the region covered by the oligonucleotide. The remaining fraction of variable genes, (6–23%, with a *Log2-ratio *< 0), showed an increased hybridisation signal of the natural strains compared to Rm 1021, suggestive of gene duplication (Table 2).

**Table 2:** Genes variable in each strain compared to strain Rm 1021

**Strain**	***Log2-ratio *> 0**	***Log2-ratio *< 0**	**Total genes variable**	Total genes analysed*	**% of variable genes**
AK58	237	50	287	6192	4.6%
AK83	273	80	353	6199	5.7%
BL225C	379	22	401	6181	6.5%
BO21CC	292	56	348	5670	6.1%

*The total number of genes analysed varies according to the number of spots discarded due to technical defects. The list of variable genes is reported as Additional Material '[see Additional file 1]'.

In order to corroborate the results of the microarray hybridisation analysis, we randomly selected 116 ORFs, with 66 of these being included in the variable ones (*P *< 0.001) and 52 found to show no significant difference compared to the type strain (table 3).

**Table 3:** Experimental analysis of 118 genes from microarray hybridisation

***P*-value classes**	**N° of ORFs**		Total ORFs amplified by PCR*	**N° of ORFs positive to PCR amplification**	**N° of ORFs analysed by Southern blotting**	N° of ORFs with duplication after Southern hybridisation**	**N° of sequenced ORFs**	**N° of ORFs with nucleotide variation in the 70-mer oligo sequence**
p < 0.001	66	log2-ratio < 0	19	19	7	7	-	-
		log2-ratio > 0	47	8	-	-	8	8
								
p > 0.001	52	log2-ratio < 0	21	21	4	0	-	-
		log2-ratio > 0	31	31	-	-	7	0

* PCR amplification was carried out with primer anchored to the flanking regions of the gene.

** Determined as higher number of observed fragments after Southern-blot analysis of Rm1021 and of the wild strain.

The 66 genes showing differential hybridisation (Table 3) were PCR amplified from genomic DNA of both strain Rm1021 and the natural isolate showing the difference. The 19 ORFs with a *Log2-ratio *< 0 selected (suggesting gene duplication) showed positive amplification. For 7 of these 19 ORFs, Southern hybridisations were carried out on restricted DNA of both tested and reference strains. All 7 ORFs showed more than one band in the DNA of wild strain compared to the single band of strain Rm 1021, confirming that the higher intensity of the microarray hybridisation of the wild strain was indeed due to a duplication of the ORF. Of the 47 ORFs with a *Log2-ratio *> 0 (indicating gene deletion or divergence), 39 gave no amplification in the wild strain, confirming the microarray result that suggested that the ORF was deleted in this strain. Eight ORFs, on the contrary, were amplified both in the wild strain and in strain Rm1021. These ORFs were sequenced and showed the occurrence of nucleotide variations in the DNA of the wild strain within the region covered by the 70-mer oligonucleotide microarray probe. In that latter case, the lower level of microarray hybridisation of the wild strains was attributed to the mismatches between the genome sequence of the natural isolate and the 70-mer probe sequence. These data confirmed the assumption made for the interpretation of the results.

We also amplified 52 ORFs randomly selected from those with a low level of probability to be variable (Table 3). All of them showed amplification from DNA of strain Rm 1021 and from DNA of the wild strain. Four of these were further analysed by Southern hybridisation showing no sign of copy number variation. Seven from the 31 ORFs with a *Log2-ratio *< 0 but with *P *> 0.001 were sequenced and no nucleotide polymorphism was observed.

### Variable genes are mainly localised on pSymA

**Figure 1:**
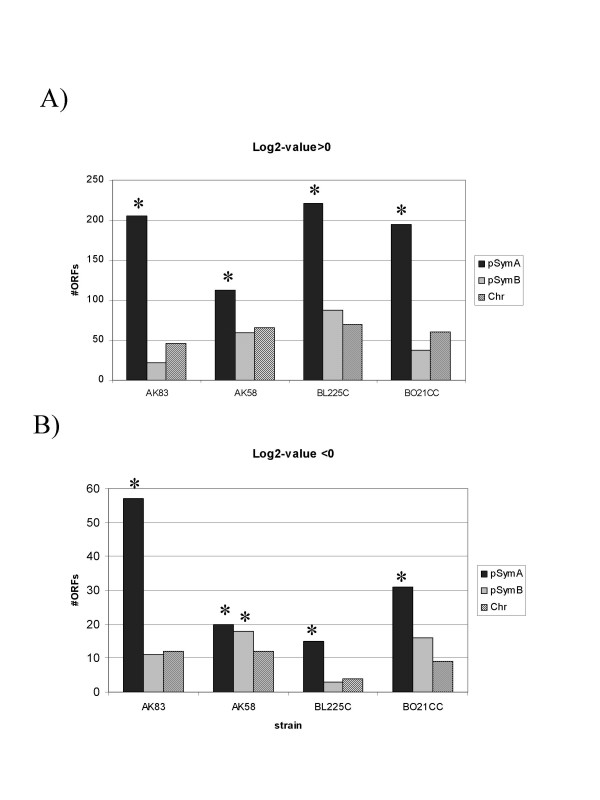
**Number and location of variable ORFs on the three replicons**. Genes considered were significantly different in hybridisation from strain Rm1021 at p < 0.001. A), Genes with *Log2-ratio *> 0; B) genes with *Log2-ratio *< 0. Asterisks over the columns indicate significant enrichment at p < 0.0001.

The genes that were found to be variable in the comparison between the type strain Rm 1021 and the four natural isolates were not randomly distributed among the three replicons. A highly significant enrichment (probability of observing this proportion <0.0001) for pSymA was found in all the strains, both for duplicated and deleted/mutated genes (Figure 1). pSymB was also found to be enriched for duplicated ORFs, though not as significantly as pSymA, except in strain AK58 where pSymB was significantly enriched for duplicated ORFs (probability of observing this proportion <0.0001).

**Figure 2:**
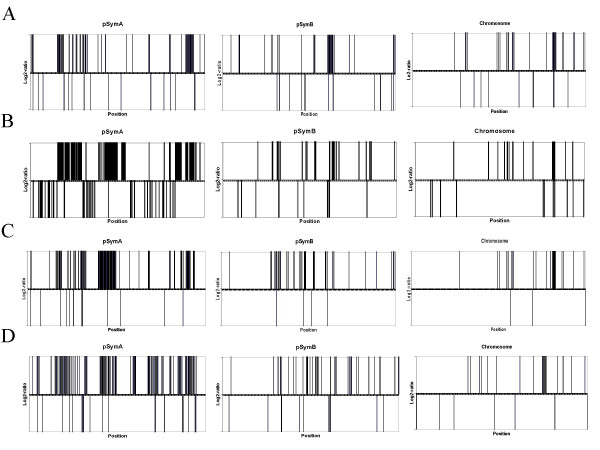
**Location of variable ORFs along the replicons**. Up and down bars indicate ORFs with *Log2-ratio *> 0 (duplication) or *Log2-ratio *< 0 (divergence or deletion), respectively. Thickness of bars indicates clusters of variable genes. A, AK58 strain; B, AK83 strain; C, BL225C strain; D, BO21CC strain. Replicon lengths are not in scale.

Within the replicons, the variable ORFs had a significant tendency to be spatially clustered (runs-test). In particular, in the pSymA plasmid, one region appeared to be duplicated in all natural strains. This region of at least 1000 bp includes two genes located upstream of the *nod*D2 gene. These genes encode the transcription factors SMa0748 and SMa0750 of the putative MucR/LysR-families. This duplication was confirmed by Southern-blot analysis on DNA extracted from strain AK83 (not shown). Among the putative deleted/mutated genes, several clusters were also identified (Figure 2)

### Functional groups of variable genes

**Figure 3:**
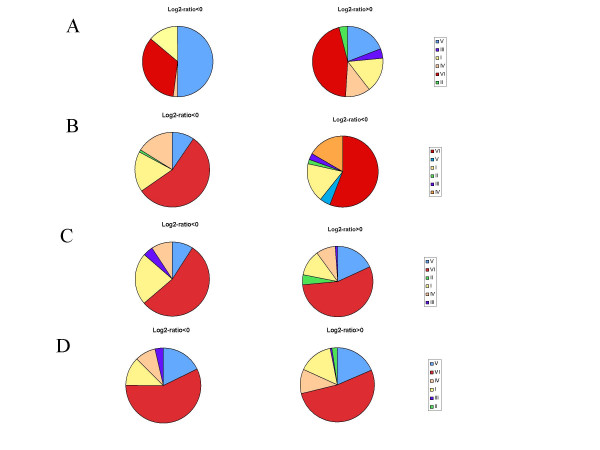
**Functional groups of variable ORFs**. A, strain AK58; B, strain AK83; C, strain BL225C; D, strain BO21CC. Classification is as defined by the S. meliloti consortium, subgroups are not reported: I, Small molecule metabolism; II, Macromolecule metabolism; III, Structural elements; IV, Cell processes; V, Elements of external origin; VI, Miscellaneous/unknown function. Groups V and VI were statistically significantly enriched for all strains.

Figure 3 reports the proportion of functional groups among the variable ORFs using the biological classification as defined by the *S. meliloti *consortium. The most frequently affected functional categories in all strains were, within the Elements of External Origin, Transposases (V.A) and, within Miscellaneous, Unknown Function ORFs (VI.D). These categories were found to be statistically significantly enriched with variable genes (see Methods).

## Discussion

The alpha-proteobacteria display diverse life-styles. In particular, they keep close relationships with the eukaryotic cell, a trait that is possibly linked to the presence in their genomes of multiple replicons [2]. In the case of the symbiotic species *Sinorhizobium meliloti*, there are three replicons, a 3.6 Mbp circular chromosome and two megaplasmids 1.3 Mbp and 1.7 Mbp in size [31,32,29].

The four strains of *S. meliloti*, whose genome have been compared in the present work with that of the type strain Rm1021, exhibited similar proportions of genes that differ in presence, nucleotide polymorphism or copy number from the type strain. The Italian strain BL225C showed the highest number of altered ORFs, while the Aral Sea strain AK58 displayed the lowest one. The overall results indicate that in the multi-replicon genome of *S. meliloti*, a fraction accounting for 4.6–6.5% of all ORFs were variable in the natural strains compared to the sequenced laboratory strain Rm1021. In particular, most of the variation was due to gene losses or to nucleotide divergence (*Log2-ratio > 0*), while a smaller fraction of the variation could be attributed to gene duplication (*Log2-ratio < 0*). These values are similar to those obtained from other studies using DNA microarrays for CGH on *Camplylobacter jejuni*, and *Staphylococcus aureus *[11,21], and are lower than those observed in human pathogens such as *Helicobacter pylori*, in which ~22% of ORFs were found to be variable [8]. However, in other *Rhizobiales*, such as *Brucella*, a similar value of gene diversity (around 4%) was found in an inter-specific analysis [23]. Of course this is a minimum amount of variation because of the unsurveyed parts of each ORF, the variation in intergenic region or new genes that are not present in the lab strain, and we therefore present a conservative estimate of genetic variation in natural strains. The variable ORFs were found to be unevenly distributed in the three replicons. The megasplasmid pSymA carried most of the variable genes. This replicon harbours *nod *genes, which are required for establishing the symbiotic relationship with host plants; *nif *genes, for nitrogen-fixation, and genes putatively involved in nitrogen and carbon metabolism and transport, as well as in stress and resistance responses, all functions intimately related to *S. meliloti*'s ecological niche [28]. Actually, the detected variable genes were found to be mainly distributed in clusters along the replicons of Rm1021. In particular for pSymA, a duplication region common to all the wild strains was found just near the *nod*D2 gene, the transcriptional activator of nodulation cascade (SMa0748-SMa0752). Moreover, pSymA contains the highest percentage of mobile genetic elements among the three *S. meliloti *replicons (3.6% for pSymA, 0.9% pSymB, 2.6% chromosome). Transposases and other related functions were particularly frequent among the variable genes. Transposable elements tend to accumulate in chromosomal regions where they do not disrupt essential cellular functions [43]. The largest amount of genetic polymorphism observed in pSymA is therefore consistent with the observation that pSymA is not essential for cell survival. Indeed, pSymA can be cured from some *S. meliloti *strains, such as Rm2011, without affecting growth in either rich or minimal-succinate media, but the cured strain is defective in the utilisation of certain carbon sources [44]. Furthermore, the analysis of the complete genome sequence of *S. meliloti *suggests that pSymA could be of foreign origin because of its lower G+C content (60.4%) and its distinct codon usage [45] compared to the other replicons. An enrichment for variable genes was also found for the pSymB megaplasmid, but only in the strain AK58. From the genomic point of view pSymB shows many features of a typical chromosome [45], carries several genes for carbohydrate metabolism and is thought to be of high adaptive value for the colonisation of soil and rhizosphere environments [2]. Since pSymA has not be mapped on the tested strains, some of the genes that hybridize on the microarray (derived from strain Rm1021) could be actually located on other replicons in the natural strains.

All functional categories of genes were represented within the variable ones (e.g., Small molecule metabolism, Macromolecule metabolism, Structural elements, Cell processes, Elements of external origin and, Miscellaneous/unknown function) with some gene found to be variable in more than one strain. However, among the different categories, the variable genes appeared to be distributed as theoretically expected from the numerical consistency of all but the last two categories. Actually, "Elements of external origin" and "Miscellaneous/unknown function" were significantly enriched in variable genes because of the large number of transposases and unknown function ORFs found to be variable. The presence of such a large proportion of unknown function genes among the polymorphic ones in natural isolates raises interesting hypotheses regarding the diversification of *S. meliloti *strains. Barnett and collaborators [28] using transcriptional profiling showed that a certain number of unknown function genes were found to be expressed below the detection threshold in both free-living culture and nodulation conditions. Several of these genes (21%, data not shown) were found in our analysis to be among the deleted ones, suggesting that they may represent pseudogenes, non-coding sequences or more interestingly, genes expressed only in very specific conditions.

## Conclusion

Using DNA microarray technology, we assessed genetic variation of the coding regions of 4 natural strains of *S. meliloti*. We found that most of the genetic differences accumulate on the symbiosis-required megaplasmid pSymA, which consequently can be considered the major hot-spot for intra-specific differentiation in *S. meliloti*.

## Methods

### Bacterial strains, microbiological media and DNA extraction

*S. meliloti *AK58 and AK83 (Table 1) are a part of alfalfa nodulating rhizobia collected by RIAM (St. Petersburg, Russia) and were trapped from soil samples collected in the Northern Aral Sea Region during May 2001 by *M. falcata*. Isolates BO21CC and BL225C, from Lodi, Italy, were trapped on *M. sativa *[34]. Rhizobia were cultured at 30°C in liquid TY medium (Tryptone 5 g/l, Yeast extract 3 g/l, CaCl_2 _0.4 g/l). DNA was extracted with the FastDNA Kit (Bio 101, Inc.) according to the manufacturer's instructions. Extracted DNA was quantified by spectrophotometric reading (Biophotometer, Eppendorf).

**Table 1:** Bacterial strains used in this study

**Strain**	**Species**	**Geographical origin**	**Host plant of isolation**
Rm 1021	*S. meliloti*	Galibert et al. 2001	Sequenced strain
AK83	*S. meliloti*	North Aral Sea, Kazakhstan	Medicago falcata
AK58	*S. meliloti*	North Aral Sea, Kazakhstan	*Medicago falcata*
BL225C	*S. meliloti*	Lodi, Italy	*Medicago sativa*
BO21CC	*S. meliloti*	Lodi, Italy	*Medicago sativa*

### PCR, Southern blot analysis and sequencing

PCR amplification reactions were performed with a Primus 96 Thermal Cycler (MWG-AG Biotech) in a 50 μl total volume with 30 ng of extracted DNA as template and contained 5 μl of 10× reaction buffer (Polytaq, Polymed, Italy), 1.5 mM MgCl_2_, 0.2 mM of each dNTP, 1 U of *Taq *DNA polymerase (Polytaq, Polymed, Italy), 10 pmols of each primer. The cycling conditions were as follows: after incubation at 95°C for 2 min, samples were cycled for 35 cycles through the following temperature profile: denaturation at 94°C for 30 sec, annealing at 57°C for 30 sec, extension at 72°C for 2 min. Finally, the mixtures were incubated at 72°C for 5 min. Then, 5 μl of each amplification mixture were analysed by agarose gel (1.2% w/v) electrophoresis in TAE buffer containing 1 μg/ml (w/v) of ethidium bromide. Southern blot analysis was performed with 1 microgram of total DNA, digested overnight at 37°C with the restriction enzymes *Xho*I, *Eco*RI or *Pvu*II, and electrophoresed for 3 h on a 0.7% agarose gel in TAE buffer with a DIG-labelled DNA marker II (Roche). DNA was blotted on a nylon membrane (Amersham). The cDNA probe preparation, the hybridisation and detection conditions were as described previously in Biondi et al. [40]. Automated DNA sequencing was performed directly from the primers used for the amplification on the purified PCR products using the BigDye Terminator v.1.1 chemistry and an ABI310 sequencer (PE-Applied Biosystems) according to the manufacturer's recommendations.

### Hybridisation and microarray scanning

Microarray slides were printed by the Center for Biotechnology, University of Bielefeld [42]. Microarrays contained 6208 70 mer oligonucleotides directed against protein-coding ORFs of *S. meliloti *1021, four 70 mer oligonucleotides directed against transgenes (gusA, lacZ, nptII, aacC1), two 70 mer stringency control oligonucleotides (80% identity), 12 alien 70 mer oligonucleotides and three alien DNA fragments (Stratagene) that can be used as spiking controls. Each microarray slide contained 6.229 triplicate spots in 48 grids of 20 rows and 21 columns. The 48 grids were arrayed in a 4 × 12 pattern of 4 metacolumns and 12 metarows. Alien oligonucleotides and 12 "housekeeping" genes were arrayed in 13 additional replicates. Oligonucleotides directed against the *S. meliloti *1021 genome and the alien oligonucleotide controls were taken from the *Sinorhizobium meliloti *Array Ready Oligo Set Version 1.0 (Qiagen).

Genomic DNA was labelled with FluoroLink Cy3- or Cy5-dCTP (Amersham Biosciences, Milano, Italy) by using the method described by Pollack et al. [46] and the components of the BioPrime DNA labeling system (Invitrogen, Milano, Italy). Two micrograms of each restriction enzyme (*Taq*I and *Msp*I) digested genomic DNA was labelled by using 20 μl of the 2.5X Random Primer, 40 U of the Klenow fragment, and 3 μl of the Cy5-dCTP or Cy3-dCTP (1 mM stocks) at 37°C for 2 h. Unincorporated fluorescent nucleotides were removed by using Microcon 30 filter columns (Millipore, Milano, Italy). The appropriate Cy5 and Cy3 labelled probes were combined and mixed with 30 μl Cot-1 DNA (1 mg/ml), 20 μl Yeast t-RNA (5 mg/ml), 450 μl TE to concentrate the samples until about 40 μl using Microcon 30 filter columns (Millipore, Milano, Italy). To each combined sample 8.5 μl of 20 × SSC and 0.74 μl of 10% SDS were added. The sample was denatured to 100°C for 1.5 min, and then incubated for 37°C for 30 min. The hybridisation probe was added to the microarray under a coverslip, and hybridisation was performed at 65°C for 16 h. Slides were washed at 60°C with 2 × SSC for 5 min and then at 60°C with 0.2 × SSC containing 0.1% SDS for 5 min and finally at room temperature with 0.2 × SSC for 2 min. The last step was conducted twice. The slides were immediately dried and scanned for fluorescence intensity by using a GenePix 4000B microarray scanner (Axon Instruments, Union City, CA), and the results were recorded in 16-bit multi-image TIFF files. Competitive hybridisation was done twice for one strain. In the first experiment, the Rm1021 reference DNA and the sample DNA from natural strain were labelled with Cy3 and Cy5, respectively. In the second hybridisation, the dyes for labelling were swapped.

For each sample a total of four slides were hybridised (after dye swapping of the two different restriction enzyme DNA preparations); considering that one slide carries three replicas of each ORF, any sample was hybridized twelve times at each ORF.

### Normalisation and significant hybridisation differences

Raw data from Genepix was imported into R (1.9) [47] and analysed using the LIMMA library (*Linear Models for Microarray Data version 1.7*, [48]). Spots showing hybridisation intensity two standard deviations above background intensity and that were not flagged as bad were used in normalisation and model fitting. For unknown reasons, strain BO21CC showed a lower number of analysable ORFs (see Table 2) as the quality of slides was apparently comparable. A within-array loess normalisation of intensities was applied. A gene was considered to have a statistically significant differences in hybridisation (moderated t-statistics using empirical Bayes shrinkage of the standard errors) when 2 of the 3 spots on the array representing that gene had a *p-value *lower than *p < 0.001*. This stringent cut-off allows preventing false positive. This analysis was designed such that positive log2 fold change occurred when hybridisation was higher in the Rm1021 strain. Such a result is indicative of sequence divergence/gene loss in other strain compared to Rm1021. Negative *Log2 *fold change occurred when more hybridisation was detected in the other strain competitively hybridised with strain Rm1021. Such a fold change pattern is indicative of gene duplication in the other strain tested compared to Rm1021.

### Physical genome location

We estimated if deleted and duplicated genes in each strain were found significantly more frequently on a given replicon. We calculated the proportion of genes associated with chromosome, megaplasmid pSymA and megaplasmid pSymB in the whole genome and then the same proportion in the significantly divergent and duplicated gene lists (p < 0.001). The hypergeometric distribution was used to calculate the probability of observing this proportion of variable genes for each replicon in comparison to their total number of genes. A Bonferroni correction was applied to adjust the cut-off probability at which a replicon is considered significantly enriched for variable genes. We multiplied the p-value by the number of tests performed and considered a replicon to be significantly enriched if this adjusted probability was below 5%.

### Spatial clustering within a replicon

Genes were binned as 1 or 0 respectively if they were differentially hybridising or not. They were then ordered according to their position along the replicons and the distribution of 1 and 0 was analysed using a runs-test [49]. This analysis tests the null hypothesis that successes in a series of binomial trials are randomly distributed. The alternative hypotheses of this test are that successes are spatially clustered or they are more evenly spaced than by chance. Genes identified as being duplicated or diverged were analysed separately.

### Functional enrichment analysis

Genes found to have a significant difference in hybridisation at a level of p < 0.001, hereafter referred to as variable genes, were used in a functional enrichment analysis. Each gene has been attributed a biological classification by the "*S. meliloti *strain Rm 1021 genome project" consortium [45]. We calculated the proportion of genes associated with each biological process in the whole genome and then in the variable gene list for each strain. The hypergeometric distribution was used to calculate the probability of observing this proportion of variable genes for each biological process in a particular strain compared to the representation in the whole genome. A Bonferroni correction for multiple testing was applied to adjust the cut-off probability at which a gene list is considered significantly enriched for a given biological classification. We multiplied the probability of observing the proportion of variable gene in a category by the number of tests performed (dependant on number of functional categories represented) and considered a gene list to be significantly enriched if this adjusted probability was below 0.05.

## Authors' contributions

EG carried out the microarray hybridizations, participated in the conceiving and in the design of the experiment and drafted the manuscript. AM carried out most of the Southern-blots and PCR verifications, participated in the conceiving and design of the experiment and drafted the manuscript. CDF and DC contributed in setting the microarray experiment protocol. NA-H and CRL performed the statistical analysis. AB contributed in providing the microarray slides and helped discussing the results. MB conceived the study, participated in its design and coordination and drafted the manuscript.

## Supplementary Material

Additional file 1 List of variable ORFs. Each column reports the list ORF's names (with p-value < 0.001) for the four different *S. meliloti *strains with Log2-ratio > 0 or <0. Click here for file
